# Cytogenomic Characterization of Murine Neuroblastoma Cell Line Neuro-2a and Its Two Derivatives Neuro-2a TR-Alpha and Neuro-2a TR-Beta

**DOI:** 10.3390/cells13221889

**Published:** 2024-11-15

**Authors:** Lioba Hergenhahn, Niklas Padutsch, Shaymaa Azawi, Ralf Weiskirchen, Thomas Liehr, Martina Rinčic

**Affiliations:** 1 Jena University Hospital, Friedrich Schiller University, Institute of Human Genetics, D-07747 Jena, Germany; lioba.hergenhahn@med.uni-jena.de (L.H.); niklas.padutsch@med.uni-jena.de (N.P.); shayma.alazawi@yahoo.com (S.A.); 2 Institute of Molecular Pathobiochemistry, Experimental Gene Therapy and Clinical Chemistry (IFMPEGKC), RWTH, University Hospital Aachen, D-52074 Aachen, Germany; rweiskirchen@ukaachen.de; 3 Croatian Institute for Brain Research, School of Medicine University of Zagreb, C-10000 Zagreb, Croatia; mrincic@hiim.hr

**Keywords:** Neuro-2a, N2A, NB2a, C1300, murine neuroblastoma cell line, multicolor banding, chromosome microarray (CMA), fluorescence in situ hybridization (FISH), satellite DNA amplification, Y-chromosome loss

## Abstract

Background: The Neuro-2a cell line, derived from a murine neuroblastoma (NB), was established as early as 1969 and originates from a transplantable tumor that arose spontaneously in an A/Jax male mouse in 1940. Since then, it has been applied in over 10,000 studies and is used by the World Organization for Animal Health for the routine diagnosis of rabies. Surprisingly, however, Neuro-2a has never been genetically characterized in detail; this study fills that gap. Methods: The Neuro-2a cell line and two of its derivatives, Neuro-2a TR-alpha and Neuro-2a TR-beta, were analyzed for their chromosomal constitution using molecular cytogenetic approaches. Array comparative genomic hybridization was performed to characterize copy number alterations. Results: Neuro-2A has a hyper-tetraploid karyotype with 70 to 97 chromosomes per cell, and the karyotypes of its two examined derivatives were quite similar. Neither of them had a Y-chromosome. The complex karyotype of Neuro-2a includes mitotically stable dicentres, neocentrics, and complex rearrangements resembling chromothripsis events. Although no amplification of euchromatin or oncogenes was detected, there are five derivative chromosomes with the amplification of centromere-near heterochromatic material and 1–5 additional derivatives consisting only of such material. Conclusions: Since satellite DNA amplification has recently been found in advanced human tumors, this finding may be the corresponding equivalent in mice. An in silico translation of the obtained results into the human genome indicated that Neuro-2A is suitable as a model for advanced human NB.

## 1. Introduction

Neuroblastoma (NB) is one of the most common solid tumors in childhood. The likelihood of developing NB varies by age, with the highest incidence in the first year of life and a decrease by age ten [1]. The range of clinical presentations includes spontaneously repressed tumors, sometimes without any treatment, to multiple metastatic diseases with a poor prognosis [1,2]. NBs originate from embryonal tumors derived from ectodermal cells that migrate from the neural crest during embryonic development and differentiate into neuronal cells. They develop from precursor cells of the sympathoadrenal lineage, responsible for forming the peripheral sympathetic nervous system and adrenal medulla [3]. As a result, most tumors occur in the area of the adrenal medulla and lumbar sympathetic ganglia [1,4].

Based on patient age, tumor extent, lymph node infiltration or distant metastasis, and the extent of initial surgical tumor resection, NBs are classified into stages according to the International Neuroblastoma Staging System (INSS). The International Neuroblastoma Risk Group Staging System (INRGSS) was developed as a further classification system, which also includes imaging-based risk factors instead of the extent of surgical resection [1,5]. The most common and prognostic adverse genetic lesion in NBs is *MYCN* gene amplification, which occurs in approximately 22% of tumors [1,4]. The gain of the long arm of chromosome 17 (17q) has emerged as one of the strongest genetic prognostic factors for survival in NBs and is predictive of a poor outcome [1]. It is the most common genetic alteration in all NBs, being present in approximately 50% of cases. Additionally, deletions in the short arm of chromosome 1 (1p), also one of the most common chromosomal alterations in NBs, are associated with an unfavorable prognosis [1]. The most frequent mechanism of 1p deletion seems to be an unbalanced translocation with 17q [1]. In tumors with *MYCN* amplification, larger deletions extending from 1p36 to the telomere occur; these are often associated with a di/tetraploid chromosome set. Furthermore, NBs with 1q, 2p gains or 3p, 4p, 11q losses are also associated with an unfavorable clinical outcome [1]. Depending on the staging and genetic characteristics, treatment ranges from purely surgical resection in low-risk groups to extensive multimodal therapy with radiotherapy, chemotherapy, autologous stem cell transplantation, and post-consolidation therapy in high-risk groups. In addition to classic chemotherapeutic agents, immunotherapeutic approaches such as the anti-GD2 antibody dinutuximab beta are also used [5].

Murine cancer cell lines are applied as a model system for human tumors, including NBs. Most of these cell lines were established decades ago. Mouse chromosomes have a more uniform shape than human ones and are difficult to characterize using banding cytogenetics, which has led to a lack of detailed genetic characterization [6] despite the widespread use of these cell lines. Neuro-2a is a murine tumor cell line derived from cells of a spontaneously developing NB of the mouse A/Jax strain in 1940 [7]; it is classified as being male-derived [8]. It was initially denominated as C1300 and was first described in 1969 [9]. The cells are neuroblasts with neuronal and amoeboid stem cell morphology. Since its establishment, the cell line has been used for countless studies, such as research into the mechanisms of proliferation and differentiation of neuronal cells, their signaling pathways, and the cytotoxic effects of chemical compounds, drugs, or toxins. For more details, see PubMed [10], where over 6000 studies are listed applying the keywords “Neuro 2a”, “Neuro-2a”, “Neuro2A”, or “Neuro2a”. Interestingly, in an additional over 1400 studies, the same cell line was denominated as “N-2a”, “N2a”, “N2A”, “Nb2a”, or “NB2a”. Finally, in another approximately 1300 studies, the line has been applied under the name “C1300”, being considered as a separate cell line from Neuro-2a in at least a dozen papers. However, ATCC clearly states for Neuro-2a that “this tumor line, designated C1300, was obtained from the Jackson Laboratory, Bar Harbor, Maine” [11]. Thus, similar results obtained for studies using Neuro-2a and C1300, for example, in a study from 2011 [12], are not surprising.

Despite being used in research for over 50 years, little is known about the chromosomal properties underlying the Neuro-2a cell line. According to ATCC, Neuro-2a has an unstable karyotype with a chromosome number between 94 and 98, containing 6–10 large, metacentric chromosomes in each cell and 2–4 “minute chromosomes” [11]. In 1989, Neuro-2a was characterized as a non-*MYCN*-amplified NB cell line [13]. That same year, a cytogenetic study on two derivatives of the C1300 cell line revealed a near-triploid karyotype [14]. In 2009, an array comparative genomic hybridization (aCGH) study was conducted on Neuro-2a, but it was not aligned with the chromosomal data [15]. In another study, Neuro-2a was karyotyped and sequenced, but the data from both approaches were not aligned by the authors, and banding cytogenetics revealed no clear results, apart from having 79 to 97 chromosomes per cell [16].

The two derivatives of the cell line Neuro-2a, Neuro-2a TR-alpha, and Neuro-2a TR-beta were also studied here, first described in 1994 [17]. Both were created by introducing a thyroid hormone receptor subunit into the genome of Neuro-2a: in the case of Neuro-2a TR-alpha, the α1 subunit of the thyroid hormone receptor, and in Neuro-2a TR-beta, the β1 subunit had a consecutive overexpression of α1 or β1 [17].

Since a comprehensive cytogenomic characterization of murine tumor cell lines can be performed using murine multicolor banding (mcb) in combination with molecular karyotyping [6,18,19], the first detailed karyotypes for the murine NB cell line Neuro-2a and its derivatives Neuro2a TR-alpha and Neuro2a TR-beta are presented. An overview of chromosomal imbalances and an in silico translation into homologous regions of the human genome is also included. 

## 2. Materials and Methods

### 2.1. Cell Lines

The murine cell line Neuro-2a was purchased from ATCC (order number: ATCC-CCL-131^TM^; Wesel, Germany), and Neuro-2a TR-alpha and TR-beta were provided by Prof. Aria Baniahmad from the Institute of Human Genetics in Jena, Germany. All cell lines were grown in adherent culture following the manufacturer’s instructions. Subsequently, tandem cytogenetic analysis and total genomic DNA extraction were performed as described below [6].

### 2.2. Chromosome Microdissection

Glass needle-based chromosome microdissection was performed as previously reported [20]. Eight centromeric regions from different murine chromosomes of a normal murine cell suspension were captured and collected in a collection drop. The DNA was then purified, amplified, and labeled with SpectrumOrange-dUTP (Abbott, Wiesbaden, Germany) using polymerase chain reaction (PCR) following the method described before [20]. A reverse FISH result on normal mouse chromosomes is depicted in Figure S1.

### 2.3. Molecular Cytogenetics

Fluorescence in situ hybridization (FISH) was conducted as previously described using whole-chromosome paints (“SkyPaintTM DNA Kit M-10 for Mouse Chromosomes”, Applied Spectral Imaging, Edingen-Neckarhausen, Germany) for multicolor-FISH (mFISH), murine chromosome-specific multicolor banding (mcb) probe mixes for FISH-banding, and the microdissection-derived probe described under Section 2.2 [6]. For each probe set, at least 30 metaphases were analyzed using Zeiss Axioplan microscopy equipped with ISIS software version 6.1.1 (MetaSystems, Altlussheim, Germany). aCGH was performed according to the standard procedures with the “SurePrint G3 Mouse CGH Microarray, 4 × 180 K” (Agilent Technologies, Waldbronn, Germany) [6].

### 2.4. Data Analyses

Imbalances and breakpoints of Neuro-2a were determined using mcb and aCGH data and aligned to human homologous regions using Ensembl and the UCSC Genome Browser (hg38/GRCh38.p13) as previously described [6] (Tables S1–S3). The data were compared to genetic alterations associated with human NBs [1] (Table 1).

### 2.5. Ethics Statement

According to the ethical committee of the medical faculty and the Animal Experimentation Commission of Friedrich Schiller University, no ethical agreements are necessary for studies involving murine tumor cell lines, including Neuro-2a and its subclones. The cell line was obtained from the Institute of Human Genetics in Jena through the American Type Culture Collection (ATCC) in the USA.

## 3. Results

**Figure 1:**
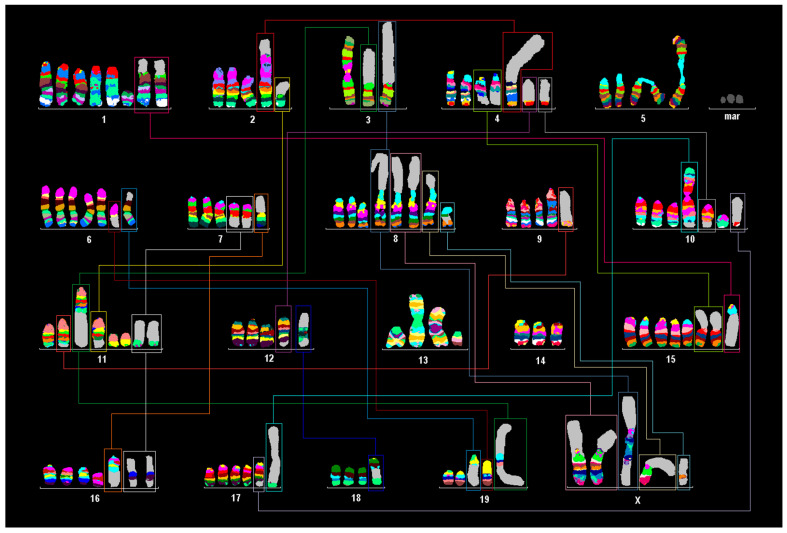
Murine multicolor banding (mcb) was applied on chromosomes of the Neuro-2a cell line. Typical pseudocolor banding for all 20 murine chromosomes is shown. This figure summarizes 20 chromosome-specific FISH experiments. Translocations are highlighted by frames in this karyogram summary.

**Figure 2:**
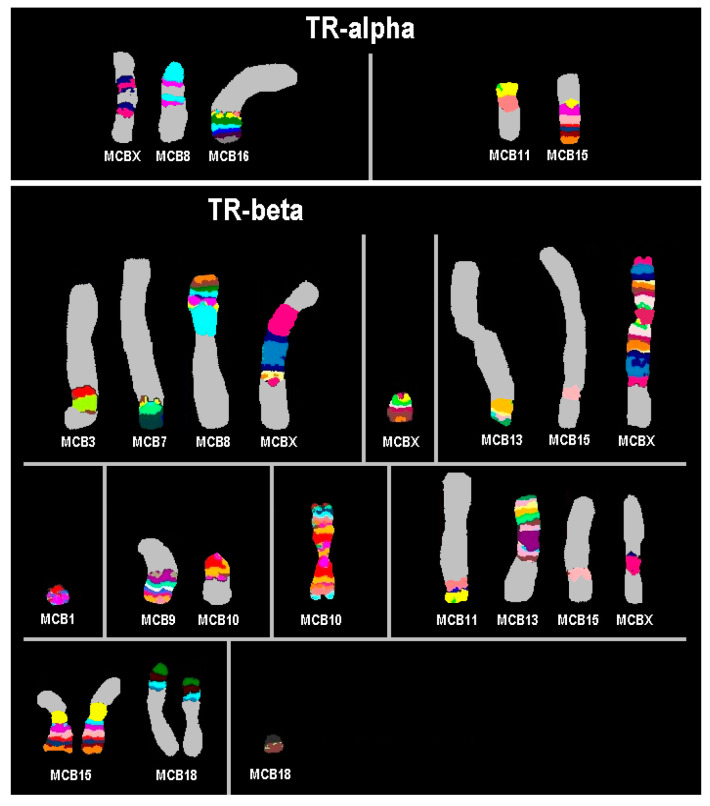
Results for Neuro-2a derivatives TR-alpha and TR-beta. Only mcb results for derivative chromosomes are shown, which were found exclusively in these two cell lines.

mFISH and mcb analyses of Neuro-2a (Figure 1), Neuro-2a TR-alpha (Figure 2), and Neuro-2a TR-beta (Figure 2) revealed that all three cell lines are closely related to each other. However, the range and percentage of chromosome numbers observed per cell varied.

**Figure 3:**
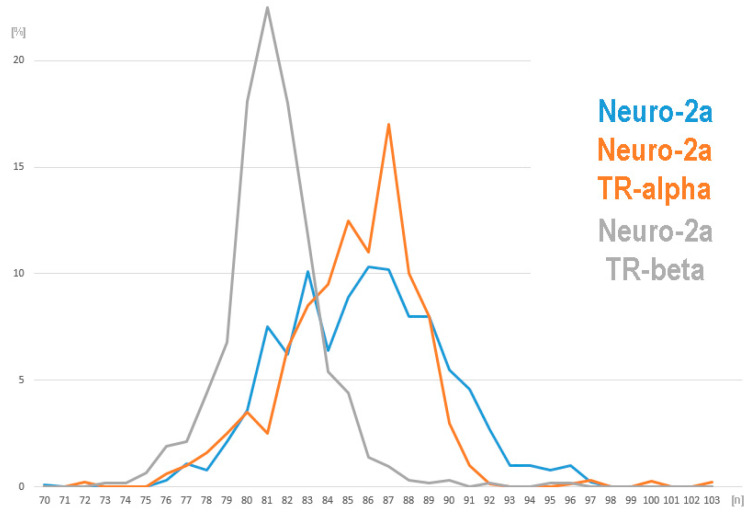
Chromosome numbers observed in 135 metaphases per cell line.

As visible in Figure 3, the ranges of the chromosomal distribution were similar in Neuro-2a and Neuro-2a TR-alpha, while the range was shifted to the left for Neuro-2a TR-beta. The average chromosome numbers were 86 chromosomes per cell for Neuro-2a, 85 chromosomes per cell for Neuro-2a TR-alpha, and 69 chromosomes per cell for Neuro-2a TR-beta.

The Neuro-2a cell line is slightly hyper-tetraploid with the following karyotype (Figure 1): 

70~97<4n>,dic(X;8)(Xqter->XA::8A1->8qter),dic(X;8)(Xqter->XA::8A1->8qter), dic(X;8)(3qter->3C::XA7->XE::XE->XA7::8A1->8qter),dic(X;8)(:XA5->XA1::8A1->8qter), +dic(X)(8pter->8A2::amp8A1::8B3.3->8C2::XA5->XA7::8C5->8qter),-Y,del(1)(G), +del(1)(G),+der(1)(pter->A5::H3->G::B->B::H6->G::B->B::H3->qter),+der(1)(pter->A5::G->H6::B->B::G->qter),+der(1)(:neo::1E->1G:),dic(2;2)(2qter->2A1::2A1->2B::4C1->4qter), idic(3)(A1;A1),-3,-3,der(4)t(4;15)(C3;D1),der(4)t(4;15)(C3;D1),idic(5)(A1;A1)(ampA1), +idic(5)(A1;A1)(ampA1),+6,+der(6)t(6;19)(A3;C1),der(7)(7pter->7D2::16C3->16C4::11D->11qter),+der(7)(7pter->7D2::16C3->16C4::11D->11qter),der(8)(pter->B3.3::C5->qter),del(9)(F1),der(9)(pter->D::A4->qter),dic(10;10)(10qter->10A1::10A1->10C3::17E5->17qter),der(10)t(4;10)(D3;B5),+der(10)(pter->A3::B5->qter),der(11)t(9;11)(F2;E2~E2),der(11)(11pter->11E2::19A~B->19D1::3A1->3qter),der(11)t(2;11)(F~G;B5),+del(11)(B2),+del(11)(B2),der(12)t(4;12)(D3;F1),idic(13)(A1;A1),dic(13;13)(qter->C2::C2->D2::neo::D2->C2::C2->qter),der(13)(:neo::B3->C1::B3->A4:),der(14)(pter->A3::D1->B::D2->qter),-14,+15,+der(15)t(1;15)(B;A1),+der(15)t(1;15)(B;A1),der(16)(pter->B5::C2->qter),+der(16)t(7;16)(D3;C1~2),der(17)t(10;17)(D1;E4),inv(17)(A3;D),der(18)(18pter->18E1::12D2->12D3 or 12A1->12A1::amp12A1::12D2->12D3 or 12A1->12A1::18E2->18qter),der(19)t(6;19) (B1;B),der(19)(amp19A1),+der(?)(A1)x1~5.

The karyotype of Neuro-2a TR-alpha is very similar to that of Neuro-2a (Figure 2). It is also slightly hyper-tetraploid with more pronounced chromosomal instability, resulting in a higher variance in chromosome numbers compared to the original parental cell line: 72~103 instead of 70~97 chromosomes per cell. The karyotype differs from that of Neuro-2a by including two new derivative chromosomes and lacking one original derivative chromosome, while one derivative is duplicated. This is illustrated here: 72~103<4n>,idem,-6,+der(8)(8pter->8B1.2::XF1->XF5::8A1->8B1.2::XF1->XF5::16B5->16qter),-der(9)(pter->D::A4->qter),+9,+der(10)t(4;10)(D3;B5),+dic(11;15)(:11B2->11A1::15A1->15qter).

The Neuro-2a TR-beta’s slightly hypo-tetraploid karyotype is more advanced than that of Neuro-2a. However, it shows a modal chromosome number varying between 73 and 96 (see also Figure 3). Compared to Neuro-2a, Neuro-2a TR-beta has nine new derivative chromosomes, lacks eleven of the derivatives present in Neuro-2a, and duplicated one of the original derivatives (Figure 2). The karyotype in relation to that of Neuro-2a is as follows: 73~96,<4n>,idem,dic(X;8)(8qter->8A1::XA7->XE::XE->XA7::3D~C->3F2::7D2->7qter),+del(X)(A5),+dic(X;X)(Xqter->XA1::XA1->XE::XB->XF5::15D1->15D3::13B->13qter),-dic(X;8)(3qter->3C::XA7->XE::XE->XA7::8A1->8qter),-dic(X;8)(:XA5->XA1::8A1->8qter),+del(1)(A5),-der(1)(:neo::1E->1G,+del(6)(G1),-der(6)t(6;19)(A3;C1),+del(9)(F1),-der(9)(pter->D::A4->qter),-der(10)t(4;10)(D3;B5),-der(10)(pter->A3::B5->qter),+der(10)t(9;10)(C;B5),+dic(10;10)(qter->B5::A3->A1::A1->A3::B5->qter),-12,-der(13)(:neo::B3->C1::B3->A4:),+dic(13;13)(13qter->13A1::13A1->13A5::XF1->XF5::15D1->15D3::11A1->11B2:),-15,-der(16)(pter->B5::C2->qter),+der(18)(18pter->18E1::cen?::15B3->15qter)x2,+neo(18)(:18E2->18qter),-der(18)(18pter->18E1::12D2->12D3 or 12A1->12A1::cen?::12D2->12D3 or 12A1->12A1::18E2->18qter),-der(19)t(6;19)(B1;B).

**Figure 4:**
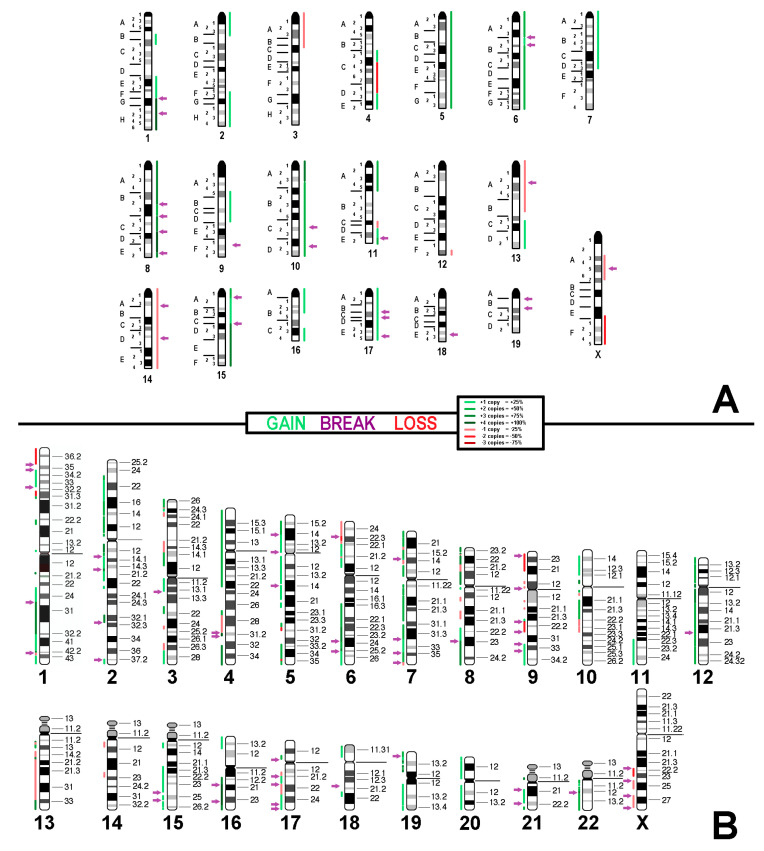
(**A**) aCGH results for the Neuro-2a cell line. Copy number alterations are depicted in a color code, with shades of red indicating losses and green indicating gains. Purple arrows point to breakpoints, which are determined based on the mcb results (**B**) The projection of the aCGH results onto the human genome highlights imbalances in specific chromosomal regions, indicating gains and losses compared to the original near-tetraploid chromosome set.

Overall, aCGH data confirmed the results of molecular cytogenetics. Data showing genomic imbalances observable in Neuro-2a are presented in Figure 4A and Table S1. Figure 4B illustrates how these imbalances translate to the human genome.

Table 1 summarizes the presence of prognostically important imbalances in human NBs compared to Neuro-2a. The aCGH data for Neuro-2a TR-alpha and TR-beta only differed marginally and could be explained in the majority of cases by a few cytogenetic alterations mentioned in the aforementioned karyotypes. These data are presented in Tables S2 and S3, as well as Figures S2 and S3.

**Figure 5:**
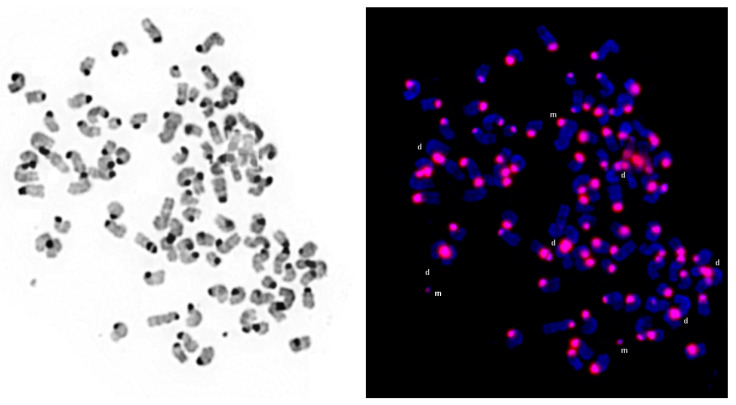
FISH results after applying the ‘all centromere probe’ for murine chromosomes on a metaphase of Neuro-2a (see also Figure S1). All six dicentric (d) and all three heterochromatic marker/derivative chromosomes (m) present in this cell are stained by this probe.

To prove that the enlargements of centromere-near sub-band A1 (which is homologous among all murine chromosomes—see Figure S1) are centromeric DNA amplifications, a corresponding microdissection-derived probe has been used in Neuro-2A. As shown in Figure 5, all 6 dicentric and all 1 to 5 heterochromatic marker chromosomes are stained by this probe.

In our study, we employed a multifaceted approach combining mFISH, mcb technique, and aCGH in the comparative cytogenomic analysis of the Neuro-2a, Neuro-2a TR-alpha, and Neuro-2a TR-beta cell lines. The methodologies allowed for a detailed visualization of chromosomal configurations and structural aberrations, revealing significant insights into the karyotype evolution of these closely related lines. Previous studies primarily relied on single-modal techniques, such as GTG-banding, mFISH, or aCGH, to analyze chromosomal anomalies, which often lack the spatial resolution and specificity that the combination of all three approaches provides.

Our results indicate that while the Neuro-2a and Neuro-2a TR-alpha cell lines exhibit similar chromosomal distributions with only minor variations in numbers, the TR-beta derivative shows marked reductions in average chromosome numbers and unique derivative formations not present in the parental and TR-alpha lines. The comparative quantitative analysis outlined in both karyotypes and Table 1 demonstrates that the TR-beta cell line’s chromosomal instability is significantly more pronounced, reinforcing our findings that extended culture periods exacerbate chromosomal variations. Additionally, the implementation of a centromere-proximal probe using microdissection-derived FISH provided novel insights into the composition of unique dicentric and marker chromosomes, suggesting a potential association with tumorigenesis processes that warrant further investigation. Thus, our comprehensive methodological integration not only corroborates previous findings but also expands our understanding of the Neuro-2a cell line’s genomic landscape, laying the groundwork for future studies exploring the implications of these chromosomal alterations in neuroblastoma biology.

## 4. Discussion

Neuro-2a is a cell line derived from a murine NB and it is known by many different names (“C1300”, “N-2a”, “N2a”, “N2A”, “Nb2a”, “NB2a”, “Neuro 2a”, “Neuro2A”, or “Neuro2a”), as highlighted in the Introduction. It has been used in approximately 10,000 published studies and is the reference cell line of the World Organization for Animal Health (OIE) for the routine diagnosis of rabies [11].

This study provides the first comprehensive cytogenomic characterization of this 55-year-old cell line [9]. Neuro-2a and its derivatives (Neuro-2a TR-alpha and Neuro-2a TR-beta) have a nearly tetraploid karyotype, consistent with ATTC [11]. However, this contradicts the claim by Sigma-Merck for the “Authenticated Neuro 2a Cell Line Sigma Aldrich”, which states that each Neuro-2a cell contains 40 chromosomes [21]. Additionally, it was confirmed that this NB cell line does not contain *MYCN* amplification. Amplification of other oncogenes was ruled out through molecular cytogenetics and aCGH, aligning with the aCGH study by Do et al. conducted in 2009 [15]. However, there are notable differences in aCGH profiles between the present study and the previous one, potentially due to the karyotype evolution of Neuro-2a over the past 15 years. In the present study, additional gains were observed in murine chromosomes 2F2-H4, 7A1-D2, 16C3.1-C4, and 17pter-qter, while in [15], not-reported losses were noted in 3A1-C, 11C-D, XA3.2-A4, XA5-A7.3, and XE3-F2.

Even though there are some differences in chromosome numbers (Figure 3) that reflect the well-known chromosome instability of cancer cells [22], the overall composite karyotypes and aCGH profiles are only slightly to moderately different when comparing Neuro-2a with Neuro-2a TR-alpha and Neuro-2a TR-beta (Figure 4, Figure S1, and Figure S2; Table S1–S3). The acquired changes are more likely due to individual karyotype evolution since their establishment 30 years ago [17] rather than the introduction of two different subunits of the thyroid hormone receptors.

The presence of near-tetraploid karyotypes in all three cell lines studied here indicates that karyotype evolution occurred in Neuro-2a. A banding cytogenetic study by Sawyer and Tuchman in 1989 revealed that 35 years ago, the cell line was only near triploid [14]. It is widely recognized that the tetraploidization of cell lines in long-term culture is a common phenomenon. In fact, in a previous study, 12 out of 25 murine tumor cell lines we examined were found to be tetraploid [19]. This suggests that polyploidization is more likely a consequence of cell culture conditions rather than a reflection of the original tumor karyotype [23]. Therefore, extended culture time is likely the main factor contributing to hyperdiploidy.

Like in previous comparable studies (summarized in [19]), Neuro-2a also has mitotically stable dicentric derivatives. This contradicts the dogma that dicentric chromosomes are always unstable and lost during cell divisions. In other murine cell lines, there was a hint of ongoing chromothripsis in a subset of the cultured cells which could not be found in Neuro-2a. However, some complex derivative chromosomes in Neuro-2a suggest the involvement of events related to chromothripsis, such as the der(1)(pter->A5::H3->G::B->B::H6->G::B->B::H3->qter) or der(1)(1pter->A5::G->H6::B->B::G->qter). The occurrence of neocentric derivatives, as present in Neuro-2a in three derivative chromosomes, was also previously seen in murine tumor cell lines [19,24].

Even though reported as a male cell line, no Y-chromosome was present in all three studied subclones of Neuro-2a. The loss of the Y-chromosome in long-term cultured murine cancer cell lines has been previously noted by us [19]. Specifically, 16 out of 25 murine tumor cell lines lost half of their sex chromosomes in culture.

Furthermore, a previously unobserved phenomenon was discovered in Neuro-2a cells. This involved a molecular cytogenetic amplification of DNA originating from the centromere-proximal sub-band A1 of various chromosomes. Specifically, this amplification was found in a dicentric complex involving chromosome X and chromosome 8 material {dic(X)(8pter->8A2::amp8A1::8B3.3->8C2::XA5->XA7::8C5->8qter)} in two isochromosomes of chromosome 5 {idic(5)(A1;A1)(ampA1)} in complex derivative 18 {der(18)(18pter->18E1::12D2->12D3 or 12A1->12A1::amp12A1::12D2->12D3 or 12A1->12A1::18E2->18qter)} in an otherwise normal chromosome 19 with an enlarged A1 region {der(19)(amp19A1)} and in one to five marker chromosomes comprising sub-band A1 from an unidentified chromosomal source. These additional chromosomes had previously been mistaken for double minutes [16]. The heterochromatic nature of these regions originating from the centromere-proximal sub-band A1 of any mouse chromosome is depicted in Figure 5. This discovery is significant, given recent findings that satellite DNA is transcribed into RNA in advanced solid tumors [25] and that centromeric satellite DNA can be amplified as homogeneously staining regions or double minutes [26]. The detection of these amplifications, which were not identifiable through aCGH but were visible through molecular cytogenetic analysis, in a murine cell line represents a potentially groundbreaking observation in this species. The presence of repetitive (likely satellite) DNA in all sub-bands A1 of murine chromosomes is illustrated in Figure 5 and Figure S1.

Finally, as highlighted in Table 1, Neuro-2a is well suited as a murine model for advanced NBs without *MYCN* amplification. Additionally, all prognostically adverse genetic factors are present in Neuro-2a (tetraploidy, loss of 1p, 3p, 11q; gain of 1q, 2p, 17q [1]); the only loss regions not observed is homologous to 4p.

In summary, our study delineates several distinct aspects that set it apart from previous research on the Neuro-2a cell line and its derivatives. Firstly, we provide the first comprehensive cytogenomic analysis that highlights not only the near-tetraploid nature of the cell lines but also the specific chromosomal gains and losses that complicate their karyotypes. This contrasts with older studies, such as the one by Do et al. in 2009 [15], which reported a more limited range of chromosomal alterations, likely due to the dynamic karyotype evolution of the Neuro-2a cell line over 15 years of culture. Furthermore, while previous work has generally relied on aCGH methodologies to elucidate chromosomal changes, our employment of both aCGH and molecular cytogenetic analyses has unveiled more nuanced aspects of karyotypic instability, including the presence of unique amplifications and neocentric derivatives that were previously undocumented. Notably, the discovery of DNA amplifications from chromosome centromere-proximal regions presents a novel perspective on genomic instability, questioning the established assumptions regarding chromosomal behavior in long-term cultured cancer cell lines. These findings not only contribute to our understanding of Neuro-2a but also emphasize the need for a multifaceted approach in cytogenomic assessments of cancer models, paving the way for future research in murine models of neuroblastoma and offering potential insight into the role of chromosomal variations in tumorigenesis.

## 5. Conclusions

In conclusion, the present study demonstrates that using the Neuro-2a cell line as a murine model of NB is justified. Therefore, the only thing that may be worth checking in the over 10,000 studies in which this cell line was used is whether it was intended as a model for advanced *MYCN* amplification-negative NB.

## Figures and Tables

**Table 1:** Comparison of copy number alterations in human NBs [1] and all three Neuro-2a cell lines.

Copy Number Aberrations in Human NBs with Adverse Prognosis (According to [1])	Copy Number Aberrations in All Three Studied Neuro-2a Cell Lines (Translated into Human Genome)
Loss of 1p	+
Gain of 1q	+
Gain of 2p	+
*NMYC* gene amplification	−
Loss of 3p	+
Loss of 4p	−
Loss of 11q	+
Gain of 17q	+

## Data Availability

All data are included in the paper.

## Supplementary Materials

The following supporting information can be downloaded at: https://www.mdpi.com/article/10.3390/cells13221889/s1, Figure S1: All centromere probe for murine chromosomes; Figure S2: aCGH results and in silico translation for the Neuro-2a TR-alpha cell line; Figure S3: aCGH results and in silico translation for the Neuro-2a TR-beta cell line; Table S1: aCGH data and its translation into the human genome for Neuro-2a; Table S2: aCGH data and its translation into the human genome for Neuro-2a TR-alpha; Table S3: aCGH data and its translation into the human genome for Neuro-2a TR-beta.
