# Supplementary material for: Cytogenomic Characterization of Murine Neuroblastoma Cell Line Neuro-2a and Its Two Derivatives Neuro-2a TR-Alpha and Neuro-2a TR-Beta
Source: Cells. 2024 Nov 15;13(22):1889. doi: 10.3390/cells13221889 (PMC11593031; doi:10.3390/cells13221889)
Supplement: Supplementary file 1 [file cells-13-01889-s001.zip › cells-3248869-supplementary.pdf]

cells

MDPI

Communication

Cytogenomic Characterization of Murine Neuroblastoma Cell Line Neuro-2a and Its Two Derivatives Neuro-2a TR-Alpha and Neuro-2a TR-Beta

Lioba Hergenhahn [1], Niklas Padutsch [1], Shaymaa Azawi [1], Ralf Weiskirchen [2], Thomas Liehr [1,*] and Martina Rinčic [3]

[1] Jena University Hospital, Friedrich Schiller University, Institute of Human Genetics, D-07747 Jena, Germany; lioba.hergenhahn@med.uni-jena.de (L.H.); niklas.padutsch@med.uni-jena.de (N.P.); shayma.alazawi@yahoo.com (S.A.)
[2] Institute of Molecular Pathobiochemistry, Experimental Gene Therapy and Clinical Chemistry (IFMPEGKC), RWTH, University Hospital Aachen, D-52074 Aachen, Germany; rweiskirchen@ukaachen.de
[3] Croatian Institute for Brain Research, School of Medicine University of Zagreb, C-10000 Zagreb, Croatia; mrincic@hiim.hr
* Correspondence: thomas.liehr@med.uni-jena.de; Tel.: +49-3641-9396850

Supplementary

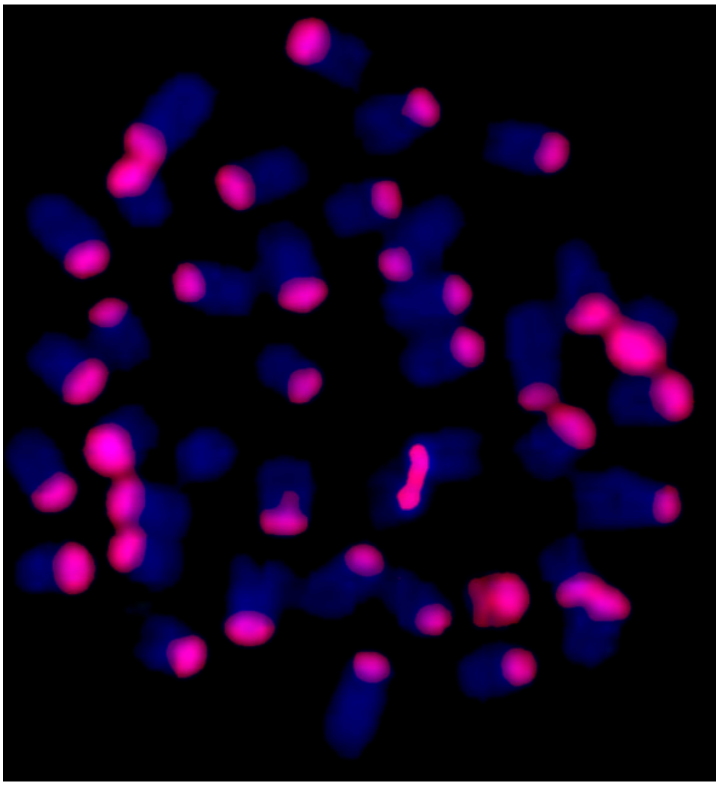

Figure S1. All centromere probe for murine chromosomes. FISH-result after applying the all centromere probe for murine chromosomes (pink) on a normal murine metaphase counterstained in blue (DAPI).

Citation: Hergenhahn, L.; Padutsch, N.; Azawi, S.; Weiskirchen, R.; Liehr, T.; Rinčic, M. Cytogenomic Characterization of Murine Neuroblastoma Cell Line Neuro-2a and Its Two Derivatives Neuro-2a TR-Alpha and Neuro-2a TR-Beta. *Cells* **2024**, *13*, 1889. https://doi.org/10.3390/cells13221889

Academic Editor: Morten Meyer

Received: 23 September 2024
Revised: 7 November 2024
Accepted: 14 November 2024
Published: date

Copyright: © 2024 by the authors. Submitted for possible open access publication under the terms and conditions of the Creative Commons Attribution (CC BY) license (https://creativecommons.org/licenses/by/4.0/).

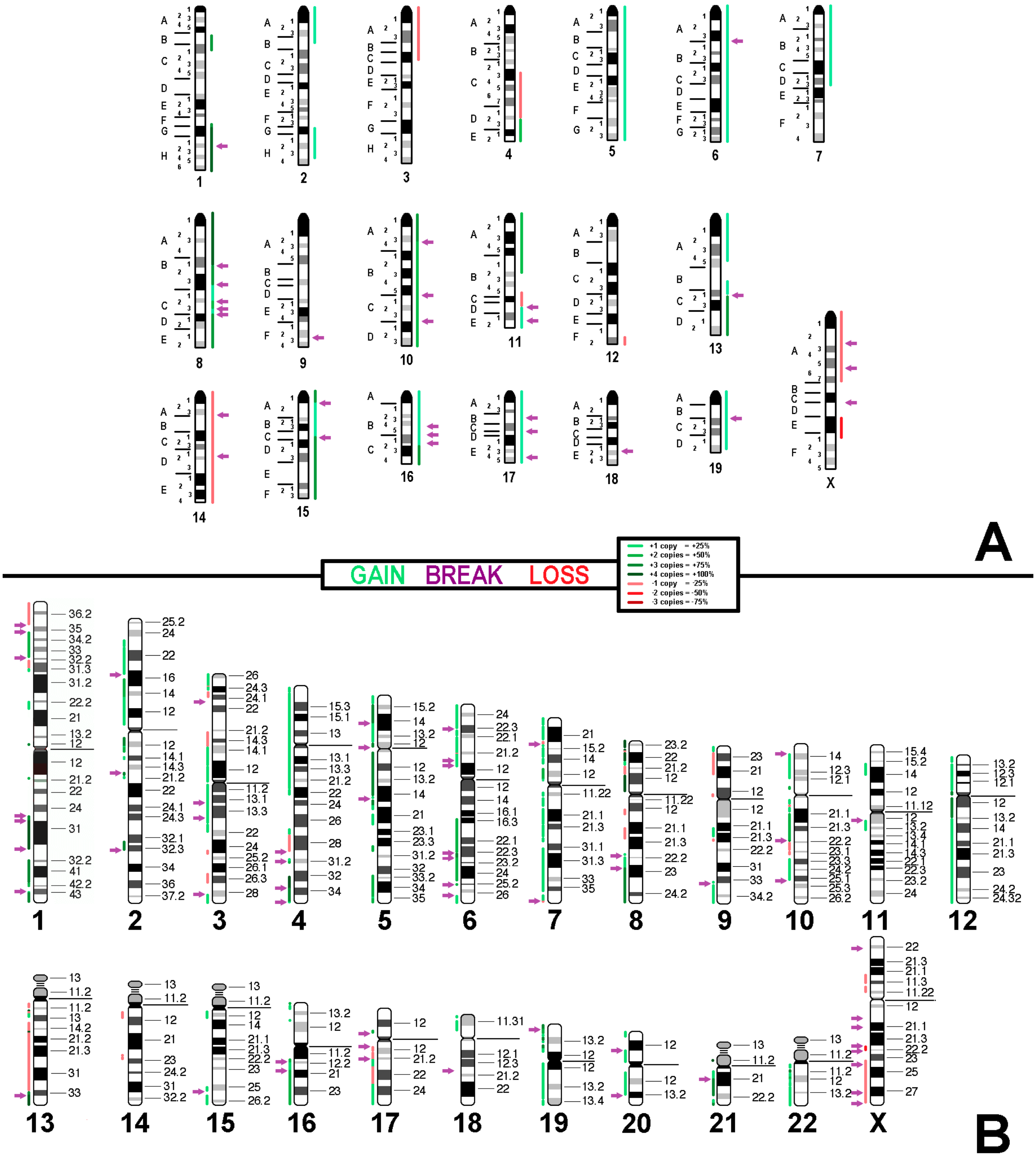

Figure S2. aCGH results and in silico translation for the Neuro-2a TR-alpha cell line. (**A**) The aCGH results for the Neuro-2a TR-alpha cell line are presented, with copy number alterations shown in a color code relative to the near-tetraploid karyotype. Losses are indicated in shades of red, gains in green, and purple arrows indicate breakpoints. Breakpoints are labeled also based on mcb results. (**B**) The aCGH results for the cell line are mapped onto the human genome, highlighting imbalances as gains and losses of specific chromosomal regions compared to the original near-tetraploid chromosome set.

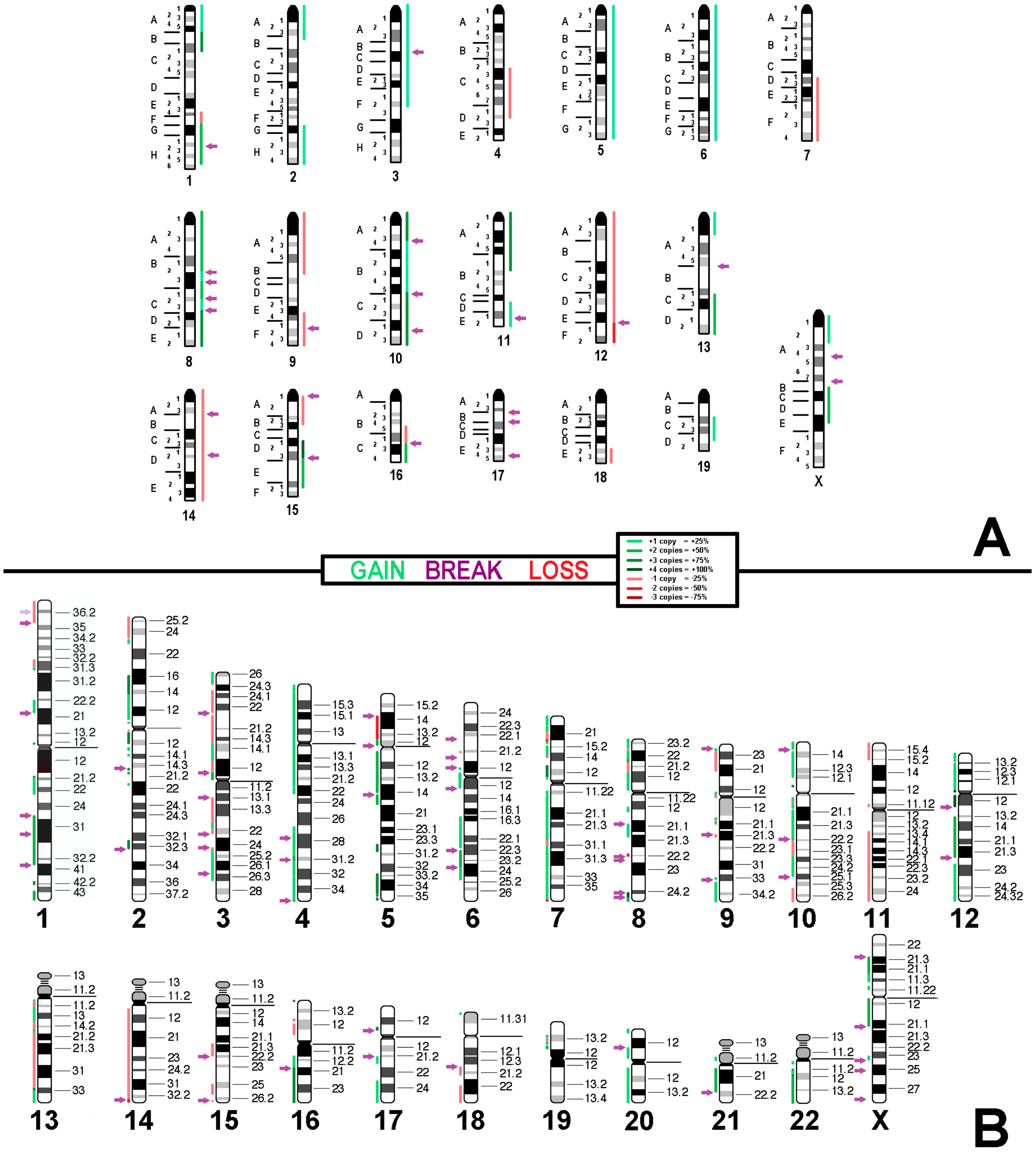

Figure S3. aCGH results and in silico translation for the Neuro-2a TR-beta cell line. (**A**) The aCGH results for the Neuro-2a TR-beta cell line display copy number alterations in relation to the near-tetraploid karyotype. The alterations are color-coded in shades of red (for losses) and green (for gains), with purple arrows indicating breakpoints. Breakpoints are labeled also according to mcb results. (**B**) The aCGH results for the cell line are projected onto the human genome, highlighting imbalances as gains and losses of specific chromosomal regions compared to the original near-tetraploid chromosome set.

Table S1. aCGH-data and its translation into the human genome for Neuro-2a. The data is given for the murine genome in GRCm38/mm10 and for humans in hg38/GRCh38.p13. The number of gains or losses with respect to the slightly hyper-tetraploid karyotype is depicted in Figure 4. The table displays regions of gains, losses and breakpoints that are visible in aCGH.

Region in Mouse		Homologous Region in Human	
**Cytobands**	**Molecular Data**	**Molecular Data**	**Cytobands**
		Gains of Copy Numbers	

1B-C1	chr1:35436704-52112433	chr2:130345670-131153454	2q21.1
		chr2:96498200-106203263	2q11.2-q12-2
		chr13:102585255-102881564	13q33.1
		chr2:188142550-189639740	2q32.1.-32-2
		chr2:189641350-194479680	2q32.2.-q32.3
1D-F	chr1:87104774-145557066	chr2:234913270-241869966	2q37.2-q37.3
		chr5:99084032-103392710	5q21.1
		chr18:60684670-67661356	18q21.32-q22.1
		chr2:121828372-125590121	2q14.3
		chr2:113678530-121820449	2q14.1-q14.3
		chr2:132380816-137850173	2q21.2-q22.1
		chr1:158547113-178469068	1q23.1-q25.2
1F-H6	chr1:145557066-195242533	chr1:178469068-207361619	1q25.2-q32.2
		chr1:240057965-246962441	1q43-q44
		chr1:207402594-227457026	1q32.2-q42.13
2A1-A3	chr2:0-32293630	chr10:5873489-15414078	10p15.1-p13
		chr10:15427950-26868173	10p13-p12.1
		chr2:137963865-138787590	2q22.1
		chr9:128309435-138124704	9q34.11-q43.3
		chr9:120763799-121365748	9q33.2
2F2-H4	chr2:128369701-qter	chr2:111847641-112892430	2q13-q14.1
		chr20:1755455-25625984	20p13-11.21
		chr20:142056-1467297	20p13
		chr20:31301793-59481159	20q11.21-q13.32
		chr20:59573167-64276082	20q13.32-q13.33
4C1-C3	chr4:63624080-72690706	chr9: 113807398-120726664	9q32-q33.2
		chr9:80378606-82087781	9q21.31-q21.32
4D2-E2	chr4:132063204-152435209	chr1:34354354-54726359	1p34.3-p32.3
5pter-qter	chr5:0-151663745	chr7:77072929-93047200	7q11.23-q21.2
		chr7:102691476-105569647	7q22.1-q22.3
		chr7:150896031-157417511	7q36.1.-q36.3
		chr7:22562781-22733045	7p15.3
		chr7:99955218-102551307	7q22.1
		chr7:149931563-150262684	7q36.1
		chr2:26171597-28801596	2p23.3-p23.2
		chr18:683166-844067	18p11.32
		chr22:31626131-32115679	22q12.2-q12.3
		chr4:1115354-3874051	4p16.3
		chr4:4183016-88079035	4p16.3-q22.1
		chr1:89498675-93369782	1p22.2-p22.1
		chr4:559243-1058871	4p16.3
		chr12:131894446-132945956	12q24,33
		chr22:24805798-28760295	22q11.23-q12.1
		chr12:107931580-110048615	12q23.3-q24.11
		chr12:110050988-121059734	12q24.11-q24.31
		chr12:121109639-131852016	12q24.31-q24.33
		chr7:55951659-56116445	7p11.2
		chr7:67371788-72722850	7q11.21-q11.23
		chr7:73145680-76520510	7q11.23
		chr7:97968996-99631744	7q21.3-q22.1
		chr7:99955218-102551307	7q22.1
		chr7:115497-6732018	7p22.3-p22.1
		chr13:26210757-33680968	13q12.13-q13.2
6pter-qter	chr6:0-149546170	chr7:93101327-97872805	7q21.2-q21.3
		chr7:7093365-12492920	7p22.1-p21.3
		chr7:112498864-128461586	7q31.1-q32.1

		chr7:128671928-149886174	7q32.1-q36.1
		chr7:150335378-150863779	7q36.1
		chr7:23214537-33063634	7p15.3-p14.3
		chr7:55364682-55573516	7p11.2
		chr4:88257546-94351949	4q22.1-q22.3
		chr4:120041924-121273532	4q27
		chr1:67166227-67851415	1p31.3
		chr2:88002903-88874857	2p11.2
		chr2:70805311-86867996	2p13.3-p11.2
		chr2:68487905-70797831	2p13.3
		chr3:126006258-129319641	3q21.2-q21.3
		chr3:12897779-15118656	3p25.2-p25.1
		chr3:64032928-75273450	3p14.1-p12.3
		chr3:12360-12059043	3p26.3-p25.2
		chr3:12075139-12855873	3p25.2
		chr3:129376089-129895684	3q21.3-q22.1
		chr10:42782538-45674259	10q11.21-q11.22
		chr12:12900-2743910	12p13.33
		chr22:17084921-18176973	22qcen-q11.21
		chr12:7905547-9061868	12p13.31
		chr12:2793954-7543294	12p13.33-p13.31
		chr12:9748769-32384500	12p13.31-p11.21
7A1-D2	chr7:0-78399952	chr19:53786804-56988760	19q13.42-q13.43
		chr19:58012427-58578185	19q13.43
		chr19:44505958-48204443	19q13.31-q13.33
		chr19:28098773-44388117	19q12-q13.31
		chr19:48296760-51418703	19q13.33-q13.41
		chr15:22476134-23039846	15q11.2
		chr15:23561415-28340921	15q11.2-q13.1
		chr15:28862278-32286393	15q13.1-q13.3
		chr15:98537156-101725667	15q26.3
		chr15:91049828-98534827	15q26.1-26.3
		chr15:85286426-88406783	15q25.3
8pter-qter	chr8:0-129106662	chr19:7112172-8069598	19p13.2
		chr13:102881565-114327455	13q33.1-q34
		chr8:397986-5501230	8p23.3-p23.2
		chr8:5510625-5890510	8p23.2
		chr8:5904979-6718069	8p23.2-p23.1
		chr13:51812906-52637583	13q14.3
		chr13:19410576-19553321	13q12.11
		chr8:36859024-42639769	8p11.23-p11.21
		chr8:29332949-36820056	8p12-p11.23
		chr8:8251254-9782907	8p23.1
		chr8:12721564-18101445	8p23.1-p22
		chr21:9594592-9809818	21p11.2
		chr4:162582872-189963502	4q32.2-q35.2
		chr8:18135309-20320465	8p22-p21.3
		chr19:16052230-19664128	19p13.12-p13.11
		chr22:33262346-35459147	22q12.3
		chr4:140330768-149971177	4q31.1-q31.23
		chr19:12627694-14572196	19p13.13-p13.12
		chr16:46659361-69942202	16q11.2-22.1
		chr16:70075624-74311815	16q22.1-q23.1
		chr1:146542324-146899900	1q21.1
		chr16:74408145-90043622	16q23.1-q24.3
		chr1:229229193-235161459	1q42.13-q42.3
		chr10:32752983-34863341	10p11.22-p11.21

9A4-D	chr9:24179425-73209432	chr7: 35028663-36454430	7p14.2
		chr11:107581891-135035473	11q22.3-q25
		chr15:51057425-51650305	15q21.2
		chr15:51669611-76221081	15q21.2-q24.2
10pter-qter	chr10:0-130495993	chr6:150073635-154676710	6q25.1-25.2
		chr6:122968765-149868599	6q22.31-25.1
		chr6:100098103-116726336	6q16.3-22.1
		chr6:116726514-122830290	6q22.1-22.31
		chr2:108449081-109621141	2q12.3-q13
		chr10:53435340-73103214	10q21.1-q22.1
		chr22:23054310-24635537	22q11.22-q11.23
		chr21:43939787-46665000	21q22.3
		chr19:14941357-15152099	19p13.12
		chr19:281181-4172053	19p13.3
		chr12:103965531-107783160	12q23.3
		chr22:32387312-33076428	22q12.3
		chr12:54962426-103957729	12q13.2-q23.3
11A1-A5~B2	chr11:0-60807373	chr22:28772603-31626130	22q12.1-q12.2
		chr7:43924481-53156756	7p13-p12.1
		chr7:53191518-55250238	7p12.1-p11.2
		chr2:53660668-68467594	2p16.2-p13.3
		chr5:173309722-174284250	5q35.2
		chr5:154952277-172505309	5q33.2-q35.1
		chr5:178104362-180869289	5q35.3
		chr5:131159221-134727937	5q23.3-q31.1
		chr5:151002149-154951429	5q33.1-q33.2
		chr1:227732052-228515511	1q42.13
		chr17:17013944-19331487	17p11.2
11D-E2	chr11:98845630-121935727	chr17:40779710-45561456	17q21.2-q21.31
		chr17:45629380-47073225	17q21.31-21.32
		chr17:47074949-47441070	17q21.32
		chr7:128466497-128616266	7q32.1
		chr17:62298466-64764269	17q23.2-q24.1
		chr17:64952801-68114549	17q24.1-q24.2
		chr17:68228066-83227287	17q24.2-q25.3
11E2	chr11:113162169-116729524	chr17:74574517-78141872	17q25.1-q25.3
13C2-D2	chr13:79347276-119475736	chr5:88393676-96808680	5q14.3-q15
		chr5:50274162-85076091	5q11.2-q14.3
		chr1:121407541-121606937	1p11.2
		chr5:43118864-45589259	5p12
15pter-qter	chr15:0-103602395	chr5:8927633-42888873	5p15.31-p12
		chr8:96434404-136853592	8q22.1-q24.23
		chr8:136870681-144619253	8q24.23-q24.3
		chr22:35566904-50784010	22.q12.3-q13.33
		chr12:33053427-34105230	12p11.21
		chr12:38213339-54655672	12q12-q13.2
16A1-B4	chr16:0-46450923	chr16:3233710-14702112	16p13.3-p13.12
		chr16:14958719-15103474	16p13.11
		chr16:15385017-16143148	16p13.11
		chr8:47293749-48952716	8q11.21
		chr12:32481985-32901827	12p11.21
		chr22:19022868-21983864	22q11.21-q11.22
		chr3:183247926-198044710	3q27.1-q29
		chr3:93808643-109113471	3q11.2-q13.13
16C3-C4	chr16:76290336-94448349	chr21:20340442-38498455	21q21.1-q22.2
17pter-qter	chr17:0-88780359	chr6:154731949-159680614	6q25.2-q25.3

		chr6:159682000-167138582	6q25.3-q27
		chr6:167442131-170584666	6q27
		chr5:96866613-99069535	5q15-q21.1
		chr16:176594-3184624	16p13.3
		chr5:172519748-173295346	5q35.1
		chr6:33500959-39090282	6p21.31-p21.2
		chr21:42070393-43703062	21q22.3
		chr19:15159485-15697397	19p13.12
		chr19:8301803-8698881	19p13.2
		chr6:29393610-33329441	6p22.1-p21.32
		chr6:39298722-49714113	6p21.2-p12.3
		chr3:16266324-20190407	3p25.1-p24.3
		chr2:106767529-108195055	2q12.3
		chr19:4229085-6940562	19p13.3-p13.2
		chr5:103423614-110727320	5q21.2-22.1
		chr18:2534402-9972544	18p11.32-p11.22
		chr2:28810654-53031720	2p23.2-p16.2
		chr18:861721-2534401	18p11.32
Loss of Copy Numbers			
3A1-C	chr3:3000029-51827766	chr8:75285702-86044133	8q21.13-q21.3
		chr8:63163338-66403590	8q12.3-q13.1
		chr3:148749748-149247516	3q24-q25.1
		chr3:168139317-183100677	3q26.2-q27.1
		chr4:121305166-139879658	4q27-q31.1
4C3-D2	chr4:72690706-123407466	chr9:82087781-83600221	9q21.32
		chr9:6847129-27300708	9p24.1-p21.2
		chr1:58654679-67096416	1p32.1-p31.3
		chr1:933238-25698616	1p36.33-p36.11
11C-D	chr11:85856951-98835348	chr17: 50781815-62248837	17q21.33-q23.2
		chr17:38195870-40769428	17q12-q21.2
12F2	chr12:116046892-120003175	chr7:157432951-159145209	7q36.3
		chr7:19713790-22489274	7p21.1-p15.3
13A1-B3	chr13:0-65308132	chr1:236023302-239921359	1q42.3-q43
		chr7:36484897-43566331	7p14.2-p13
		chr6:20064992-28544283	6p22.3-p22.1
		chr6:181261-17755386	6p25.3-p22.3
		chr6:17758154-20060567	6p22.3
		chr9:88426259-94305430	9q22.1-q23.32
		chr5:174325424-177612610	5q35.2-q35.3
		chr5:134737788-137755249	5q31.1-q31.2
		chr9:83617040-87725484	9q21.32-21.33
		chr9:94558675-96664521	9q22.32-q22.33
		chr9:38669564-39362991	9p13.1-p12
		chr9:61321216-61453115	9q12
		chr9:66985272-67553332	9q21.11
		chr9:41807600-42190916	9p11.2
14pter-qter	chr14:7756543-124743915	chr3:57956447-64024024	3p14.3-p14.1
		chr3:23104895-27667234	3p24.3-p24.1
		chr14:51805337-52106937	14q22.1
		chr6:39101990-39298710	6p21.2
		chr10:73110406-79495343	10q22.2-q22.3
		chr10:80053353-80210587	10q22.3
		chr3:52316044-57945503	3p21.1-p14.3
		chr3:15203607-16266323	3p25.1
		chr10:45868532-49967632	10q11.22-11.23
		chr10:80261810-87216559	10q23.1-q23.2

		chr14:52221917-58163176	14q22.1-q23.1
		chr14:19735448-22100195	14q11.2
		chr14:22132047-24680753	14q11.2-q12
		chr13:24761821-24937784	13q12.12-q12.13
		chr13:19633139-22705235	13q12.11-q12.12
		chr13:49247854-49587268	13q14.2
		chr13:25025580-26075099	13q12.13
		chr13:23279259-24322217	13q12.12
		chr13:49620101-51782351	13q14.2-q14.3
		chr8:9812600-11884194	8p23.1
		chr8:20349073-29293682	8p21.3-p12
		chr13:40895805-49224923	13q14.11-q14.2
		chr13:52651898-102437231	13q14.3-q33.1
XA3-A4	chrX:35708989-45953111	chrX:118452702-127335026<br>chrX:127404034-127683649	Xq24-q25
XA5-A7	chrX:57430731-73817486	chrX:137117025-153503780	Xq26.3-q28
XE3-F2	chrX:131664614-147756703	chrX:99351967-115283323	Xq22.1-q23
All Breakpoints Observed			
1B	chr1:35436704	chr2:130345670	2q21.1
1C1	chr1:52112433	chr2:194479680	2q32.3
1D	chr1:87104774	chr2:234913270	2q37.2
1F	chr1:145557066	chr1:178469068	1q25.2
2B	chr2:32293630	chr9:121365748	9q33.2
2F2	chr2:128369701	chr2:111847641	2q13
3C	chr3:51827766	chr4:139879658	4q31.1
4C1	chr4:63624080	chr9:113807398	9q32
4C3	chr4:72690706	chr9:82087781	9q21.32
4D2	chr4:123407466	chr1:25698616	1p36.11
4D2	chr4:132063204	chr1:34354354	1p34.3
4E2	chr4:152435209	chr1:54726359	1p32.3
6A3	chr6:27663441	chr7:126845785	7q31.33
6B2	chr6:41545199	chr7:142882659	7q34
6G3	chr6:149546170	chr12:32384500	12p11.21
7D2	chr7:78399952	chr15:88406783	15q25.3
8B3	chr8:72823474	chr22:33124372	22q12.3
8C2	chr8:80066496	chr4:146571444	4q31.22
8C5	chr8:94536540	chr16:56919767	16q13
8E1	chr8:114569098	chr16:81075933	16q23.2
9A4	chr9:24179425	chr7:35028663	7p14.2
9D	chr9:73209432	chr15:76221081	15q24.2
10A3	chr10:23524569	chr6:139744376	6q24.1
10C1	chr10:80168862	chr19:2743029	19p13.3
10D2	chr10:117758252	chr12:91353454	12q21.33
11B2	chr11:60807373	chr17:19331487	17p11.2
11C	chr11:85856951	chr17:50781815	17q21.33
11D	chr11:98845630	chr17:40779710	17q21.2
11E2	chr11:113162169	chr17:74574517	17q25.3
11E2	chr11:116729524	chr17:78141872	17q25.3
11E2	chr11:121935727	chr17:83227287	17q25.3
13A4	chr13:44257895	chr6:15266674	6p22.3
13B3	chr13:65308132	chr9:67553332	9q22.33
13C2	chr13:79347276	chr5:88393676	5q14.3
13D2	chr13:119475736	chr5:45589259	5p12
15A1	chr15:10830999	chr5:20960482	5p14.3
15B3	chr15:41535352	chr8:108092542	8q23.1

16B4	chr16:46450923	chr3:109113471	3q13.13
16C3	chr16:76290336	chr21:20340442	21q21.1
16C4	chr16:94448349	chr21:38498455	21q22.2
18E2	chr18:72495328	chr18:49093539	18q21.1
19C1	chr19:28848069	chr9:5319499	9p24.1
XA3	chrX:35708989	chrX:118452702	Xq24
XA4	chrX:45953111	chrX:127404034	Xq25
XA5	chrX:57430731	chrX:137117025	Xq26.3
XA7	chrX:73817486	chrX:153503780	Xq28
XE3	chrX:131664614	chrX:99351967	Xq22.1
XF2	chrX:147756703	chrX: 115283323	Xq23

Table S2. aCGH-data and its translation into human genome for Neuro-2a TR-alpha. The data is given for the murine genome in GRCm38/mm10 and for the human genome in hg38/GRCh38.p13. The number of gains or losses relative to the slightly hyper-tetraploid karyotype is presented in Figure S2. The table displays regions of gains, losses and breakpoints that are visible in aCGH.

**Region in Mouse**		**Homologous Region in Human**	
**Cytobands**	**Molecular Data**	**Molecular Data**	**Cytobands**
	Gains of Copy Numbers		
1B-C1	chr1:35400258-52112433	chr2:130309224-131153454	2q21.1
		chr2:96498200-106203263	2q11.2-2q12.2
		chr13:102585255-102881564	13q33.1
		chr2:188142550-189639740	2q32.1-2q32.2
		chr2:189641350-194479680	2q32.2-2q32.3
1G-H2	chr1:150809088-167362738	chr1:183721090-200274740	1q25.3-q32.1
1F-H6	chr1:145557066-195221225	chr1:178469068-207361619	1q25.2-q32.2
		chr1:240057965-246962441	1q43-q44
		chr1:207402594-227457026	1q32.2-q42.13
2A1-B	chr2:0-32282208	chr10:6819812-15414078	10p14-p13
		chr10:15427950-26868173	10p13-p12.1
		chr10:27110043-27242311	10p12.1
		chr2:112974505-113379867	2q14.1
		chr9:128309435-138124704	9q34.11-q34.3
		chr9:120763799-121354326	9q33.2
2G1-H3	chr2:142054244-168401112	chr20:16638826-25625984	20p12.1-p11.21
		chr20:142056-1467297	20p13
		chr20:31301793-52967732	20q11.21-q13.2
4D2-E2	chr4:132063204-152548484	chr1:34354354-54839634	1p34.2-p32.3
5pter-qter	chr5:3247009-151663745	chr7:77072929-93047200	7q11.23-q21.2
		chr7:102691476-105569647	7q22.1-q22.3
		chr7:22811944-23201057	7p15.3
		chr7:150896031-157417511	7q36.1-q36.3
		chr7:22562781-22733045	7p15.3
		chr2:26171597-28801596	2p23.3-p23.2
		chr18:683166-844067	18p11.32
		chr22:31626131-32115679	22q12.2-q12.3
		chr4:1115354-3874051	4p16.3
		chr4:4183016-88079035	4p16.3-q22.1
		chr1:89498675-93369782	1p22.2-22.1
		chr4:559243-1058871	4p16.3
		chr12:131894446-132945956	12q24.33
		chr22:24805798-28760295	22q11.23-q12.1
		chr12:107931580-110048615	12q23.3-q24.11
		chr12:110050988-121059734	12q24.11-q24.31
		chr12:121109639-131852016	12q24.31-q24.33

		chr7:55951659-56116445	7p11.2
		chr7:67371788-72722850	7q11.21-q11.23
		chr7:73145680-76520510	7q11.23
		chr7:99955218-102551307	7q22.1
		chr7:115497-6732018	7p22.3-p22.1
		chr7:97968996-99631744	7q21.3-q22.1
		chr13:26210757-33680968	13q12.13-q13.2
6pter-qter	chr6:0-149546170	chr7:93101327-97872805	7q21.2-q21.3
		chr7:7093365-12492920	7p22.1-p21.3
		chr7:112498864-128461586	7q31.1-q32.1
		chr7:128671928-149886174	7q32.1-q36.1
		chr7:150335378-150863779	7q36.1
		chr7:23214537-33063634	7p15.3-p14.3
		chr7:55364682-55573516	7p11.2
		chr4:88257546-94351949	4q22.1-q22.3
		chr4:120041924-121273532	4q27
		chr1:67166227-67851415	1p31.3
		chr2:88002903-88874857	2p11.2
		chr2:70805311-86867996	2p13.3-p11.2
		chr2:68487905-70797831	2p13.3
		chr3:126006258-129319641	3q21.2-q21.3
		chr3:12897779-15118656	3p25.2-p25.1
		chr3:64032928-75273450	3p14.1-p12.3
		chr3:12360-12059043	3p26.3-p25.2
		chr3:12075139-12855873	3p25.2
		chr3:129376089-129895684	3q21.3-q22.1
		chr10:42782538-45674259	10q11.21-q11.22
		chr12:12900-2743910	12p13.33
		chr22:17084921-18176973	22qcen-q11.21
		chr12:7905547-9061868	12p13.31
		chr12:2793954-7543294	12p13.33-p13.31
		chr12:9748769-32384500	12p13.31-p11.21
7A1-D2	chr7:0-78399952	chr19:53786804-56988760	19q13.42-q13.43
		chr19:58012427-58578185	19q13.43
		chr19:44505958-48204443	19q13.31-q13.33
		chr19:28098773-44388117	19q12-13.31
		chr19:48296760-51418703	19q13.33-q13.41
		chr16:16158958-16294817	16p13.11
		chr11:17381938-25229599	11p15.1-p14.3
		chr15:22476134-23039846	15q11.2
		chr15:28570709-28682263	15q13.1
		chr15:23561415-28340921	15q11.2-q13.1
		chr15:28862278-32286393	15q13.1-13.3
		chr15:98537156-101725667	15q26.3
		chr15:91049828-98534827	15q26.1-26.3
		chr15:85286426-88406783	15q25.3
8pter-qter	chr8:0-129106662	chr19:7112172-8069598	19p13.2
		chr13:102881565-114327455	13q33.1-q34
		chr8:397986-5501230	8p23.3-p23.2
		chr8:5510625-5890510	8p23.2
		chr8:5904979-6718069	8p23.2-p23.1
		chr13:51812906-52637583	13q14.3
		chr13:19410576-19553321	13q12.11
		chr8:36859024-42639769	8p11.23-p11.21
		chr8:29332949-36820056	8p12-p11.23
		chr8:8251254-9782907	8p23.1
		chr8:12721564-18101445	8p23.1-p22

		chr21:9594592-9809818	21p11.2
		chr4:162582872-189963502	4q32.2-q35.2
		chr8:18135309-20320465	8p22-p21.3
		chr19:16052230-19664128	19p13.12-p13.11
		chr22:33262346-35459147	22q12.3
		chr4:140330768-149971177	4q31.1-q31.23
		chr19:12627694-14572196	19p13.13-p13.12
		chr16:46659361-69942202	16q11.2-22.1
		chr16:70075624-74311815	16q22.1-q23.1
		chr1:146542324-146899900	1q21.1
		chr16:74408145-90043622	16q23.1-q24.3
		chr1:229229193-235161459	1q42.13-q42.3
		chr10:32752983-34863341	10p11.22-p11.21
10A1-C1	chr10:5967563-80148648	chr6:153309809-154676710	6q25.2
		chr6:122968765-149868599	6q22.31-q25.1
		chr6:100098103-116726336	6q16.3-q22.1
		chr6:116726514-122830290	6q22.1-q22.31
		chr2:108449081-109621141	2q12.3-q13
		chr10:53435340-73103214	10q21.1-q22.1
		chr22:23054310-24635537	22q11.22-q11.23
		chr21:43939787-46665000	21q22.3
		chr19:14941357-15152099	19p13.12
		chr19:281181-2722815	19p13.3
10pter-qter	chr10:0-130495993	chr6:150073635-154676710	6q25.1-25.2
		chr6:122968765-149868599	6q22.31-25.1
		chr6:100098103-116726336	6q16.3-22.1
		chr6:116726514-122830290	6q22.1-22.31
		chr2:108449081-109621141	2q12.3-q13
		chr10:53435340-73103214	10q21.1-q22.1
		chr22:23054310-24635537	22q11.22-q11.23
		chr21:43939787-46665000	21q22.3
		chr19:14941357-15152099	19p13.12
		chr19:281181-4172053	19p13.3
		chr12:103965531-107783160	12q23.3
		chr22:32387312-33076428	22q12.3
		chr12:54962426-103957729	12q13.2-q23.3
11A1-B2	chr11:3207002-60788781	chr22:28772603-31626130	22q12.1-q12.2
		chr7:43924481-53156756	7p13-p12.1
		chr7:53191518-55250238	7p12.1-p11.2
		chr2:53660668-68467594	2p16.2-p14
		chr5:173309722-174284250	5q35.2
		chr16:28819-173821	16p13.3
		chr5:154952277-172505309	5q33.2-q35.1
		chr5:178104362-180869289	5q35.3
		chr5:131159221-134727937	5q23.3-q31.1
		chr5:151002149-154951429	5q33.1-q33.2
		chr1:227732052-228515511	1q42.13
		chr17:17013944-19126397	17p11.2
11D-E2	chr13:79347276-119475736	chr17:40779710-45561456	17q21.2-q21.31
		chr17:45629380-47073225	17q21.31-21.32
		chr17:47074949-47441070	17q21.32
		chr7:128466497-128616266	7q32.1
		chr17:62298466-64764269	17q23.2-q24.1
		chr17:64952801-68114549	17q24.1-q24.2
		chr17:68228066-83227287	17q24.2-q25.3
13A1-A5	chr13:14145531-44334215	chr1:239867374-239921359	1q43
		chr7:36484897-43566331	7p14.2-p13

		chr6:20064992-28544283	6p22.3-p22.1
		chr6:181261-17755386	6p25.3-p22.3
13B3-C1	chr13:66880741-76256890	chr8:96234800-96361600	8q22.1
		chr5:191310-7935328	5p15.33-p15.31
		chr5:85240523-85303290	5q14.3
13C2-D2	chr13:79486537-120264543	5q14.3-q15	chr5:88393676-96808680
		5q11.2-q14.3	chr5:50274162-85076091
		1p11.2	chr1:121407541-121606937
		5p12	chr5:43118864-45589259
15pter-qter	chr15:0-103602395	chr5:8927633-42888873	5p15.31-p12
		chr8:96434404-136853592	8q22.1-q24.23
		chr8:136870681-144619253	8q24.23-q24.3
		chr22:35566904-50784010	22.q12.3-q13.33
		chr12:33053427-34105230	12p11.21
		chr12:38213339-54655672	12q12-q13.2
16pter-qter	chr16:0-94448349	chr16:3233710-14702112	16p13.3-p13.12
		chr16:14958719-15103474	16p13.11
		chr16:15385017-16143148	16p13.11
		chr8:47293749-48952716	8q11.21
		chr12:32481985-32901827	12p11.21
		chr22:19022868-21983864	22q11.21-q11.22
		chr3:183247926-198044710	3q27.1-q29
		chr3:93808643-125624615	3q11.2-q21.2
		chr3:75817777-90260450	3p12.3-p11.2
		chr2:131846708-132000018	2q21.2
		chr21:14143207-38498455	21q11.2-q22.2
17pter-qter	chr17:0-88780359	chr6:154731949-159680614	6q25.2-q25.3
		chr6:159682000-167138582	6q25.3-q27
		chr6:167442131-170584666	6q27
		chr5:96866613-99069535	5q15-q21.1
		chr16:176594-3184624	16p13.3
		chr5:172519748-173295346	5q35.1
		chr6:33500959-39090282	6p21.31-p21.2
		chr21:42070393-43703062	21q22.3
		chr19:15159485-15697397	19p13.12
		chr19:8301803-8698881	19p13.2
		chr6:29393610-33329441	6p22.1-p21.32
		chr6:39298722-49714113	6p21.2-p12.3
		chr3:16266324-20190407	3p25.1-p24.3
		chr2:106767529-108195055	2q12.3
		chr19:4229085-6940562	19p13.3-p13.2
		chr5:103423614-110727320	5q21.2-22.1
		chr18:2534402-9972544	18p11.32-p11.22
		chr2:28810654-53031720	2p23.2-p16.2
		chr18:861721-2534401	18p11.32
19pter-qter	chr19:0-5982459	chr11:61061633-68942254	11q12.2-q13.3
		chr9:68221841-80162449	9q21.11-q21.31
		chr2:113413562-113564048	2q14.1
		chr9:51702-6659223	9p24.3-p24.1
		chr10:50157843-52780322	10q11.23-q21.1
		chr10:87474356-107971641	10q23.2-q25.1
Loss of Copy Numbers			
3A1-C	chr3:0-51827766	chr8:75285702-86044133	8q21.13-q21.3
		chr8:63163338-66403590	8q12.3-q13.1
		chr3:148749748-149247516	3q24-q25.1
		chr3:168139317-183100677	3q26.2-q27.1

		chr9:39883273-40101748	9p12-p11.2
		chr4:121305166-139879658	4q27-q31.1
4C3-D2	chr4:72690706-123407466	chr9:82087781-83600221	9q21.32
		chr9:6847129-27300708	9p24.1-p21.2
		chr1:58654679-67096416	1p32.1-31.3
		chr1:933238-25698616	1p36.33-p36.11
11B5-D	chr11:79334813-98835348	chr17:32247818-37831987	17q11.2-q12
		chr17:47482968-62248837	17q21.32-q23.2
		chr17:38195870-40769428	17q12-q21.2
12F2	chr12:115794872-120003175	chr7:19713790-22464085	7q36.3
14pter-qter	chr14:7756543-124743915	chr3:57956447-64024024	3p14.3-p14.1
		chr3:23104895-27667234	3p24.3-p24.1
		chr14:51805337-52106937	14q22.1
		chr6:39101990-39298710	6p21.2
		chr10:73110406-79495343	10q22.2-q22.3
		chr10:80053353-80210587	10q22.3
		chr3:52316044-57945503	3p21.1-p14.3
		chr3:15203607-16266323	3p25.1
		chr10:45868532-49967632	10q11.22-11.23
		chr10:80261810-87216559	10q23.1-q23.2
		chr14:52221917-58163176	14q22.1-q23.1
		chr14:19735448-22100195	14q11.2
		chr14:22132047-24680753	14q11.2-q12
		chr13:24761821-24937784	13q12.12-q12.13
		chr13:19633139-22705235	13q12.11-q12.12
		chr13:49247854-49587268	13q14.2
		chr13:25025580-26075099	13q12.13
		chr13:23279259-24322217	13q12.12
		chr13:49620101-51782351	13q14.2-q14.3
		chr8:9812600-11884194	8p23.1
		chr8:20349073-29293682	8p21.3-p12
		chr13:40895805-49224923	13q14.11-q14.2
		chr13:52651898-102437231	13q14.3-q33.1
XA1-A7	chrX:5950887-73835695	chrX:48455475-51616130	Xp11.23-p11.22
		chrX:37491877-47660779	Xp21.1-p11.23
		chrX:115988254-118451148	Xq23-q24
		chrX:118452702-127335026	Xq24-q25
		chrX:127404034-153521989	Xq25-q28
XE-F1	chrX:131664614-136315994	chrX:99351967-104003347	Xq22.1-q22.2
	All Breakpoints Observed		
1B	chr1:35400258	chr2:130309224	2q21.1
1C1	chr1:52112433	chr2:194479680	2q32.3
1F	chr1:145557066	chr1:178469068	1q25.2
1G	chr1:150809088	chr1:183721090	1q25.2
1H2	chr1:167362738	chr1:200274740	1q32.1
2B	chr2:32282208	chr9:121354326	9q33.2
2G1	chr2:142054244	chr20:16638826	20p12.1
2H3	chr2:168401112	chr20:52967732	20q13.2
3C	chr3:51827766	chr4:139879658	4q31.1
4C3	chr4:72690706	chr9:82087781	9q21.32
4D2	chr4:123407466	chr1:25698616	1p36.11
4D2	chr4:132063204	chr1:34354354	1p34.2
4E2	chr4:152548484	chr1:54839634	1p32.3
7D2	chr7:78399952	chr15:88406783	15q25.3
8A1	chr8:5568439	chr13:106013751	13q33.1
8B1	chr8:48527372	chr4:171093529	4q34.1

8B3	chr8:66076700	chr4:188642857	4q35.2
8C2	chr8:80480603	chr4:146985551	4q31.22
8C3	chr8:85816153	chr16:48199380	16q12.1
8D1	chr8:94430045	chr16:56813272	16q12.1
9F3	chr9:112170723	chr3:30900479	3p23
10A1	chr10:4378977	chr6:153309809	6q25.2
10A3	chr10:21776096	chr6:138276097	6q23.3
10C1	chr10:80148648	chr19:2722815	19p13.3
10C1	chr10:74406721	chr10:72596909	10q22.1
11B2	chr11:60788781	chr17:19126397	17p11.2
11B5	chr11:79334813	chr17:32247818	17q11.2
11D	chr11:98845630	chr17:40779710	17q21.2
11D	chr11:98835348	chr17:40769428	17q21.2
12F2	chr12:115794872	chr7:19713790	7q36.3
13A1	chr13:14145531	chr1:239867374	1q43
13A5	chr13:44334215	chr6:17755386	6p22.3
13B3	chr13:66880741	chr8:96234800	8q22.1
13C1	chr13:76256890	chr5:85303290	5q14.3
13C2	chr13:79347276	chr5:88393676	5q14.3
13D2	chr13:119475736	chr5:45589259	5p12
15A1	chr15:10830999	chr5:20960482	5p14.3
15B3	chr15:41535352	chr8:108092542	8q23.1
16B1	chr16:25578204	chr3:190494533	3q28
16B5	chr16:48703443	chr3:111365991	3q13.13
16C1	chr16:58711023	chr3:121373571	3q13.33
16C3	chr16:76383775	chr21:20433881	21q21.1
16C4	chr16:94448349	chr21:38498455	21q22.2
17B.3	chr17:44537565	chr6:44309305	6p21.1
17C	chr17:47723244	chr6:47494984	6p12.3
18E2	chr18:70930568	chr18:47528779	18q21.1
XA2	chrX:24072117	chrX:118451148	Xq24
XA6	chrX:63990580	chrX:143676874	Xq27.3
XA7	chrX:73835695	chrX:153521989	Xq28
XC3	chrX:99993286	chrX:71072698	Xq13.1
XD	chrX:109102727	chrX:80182139	Xq21.1
XE	chrX:131664614	chrX:99351967	Xq22.1
XF1	chrX:136315994	chrX:104003347	Xq22.2
XF4	chrX:162556216	chrX:16397589	Xp22.2

Table S3. aCGH-data and its translation into the human genome for Neuro-2a TR-beta. The data is given for the murine genome in GRCm38/mm10 and for humans in hg38/GRCh38.p13. The number of gains or losses in relation to the nearly tetraploid karyotype is given in Figure S3. The table displays regions of gains, losses and breakpoints that are visible in aCGH.

**Region in Mouse**		**Homologous Region in Human**	
**Cytobands**	**Molecular Data**	**Molecular Data**	**Cytobands**
Gains of Copy Numbers			
1A4-A5	chr1:20494109-29328338	chr6:51899790-52703905	6p12.2
		chr6:56359076-68127998	6p12.1-q12
1B-C1	chr1:35737073-52112433	chr2:130646039-131153454	2q21.1
		chr2:96498200-106203263	2q11.2-q12.2
		chr13:102585255-102881564	13q33.1
		chr2:188142550-189639740	2q32.1-q32.2
		chr2:189641350-194479680	2q32.2-q32.3
1F-H5	chr1:145557066-182502146	chr1:178469068-207361619	1q25.2-q32.2
		chr1:240057965-246962441	1q43-q44

		chr1:207402594-214738082	1q32.2-q41
2A1-B	chr2:0-32293689	chr10:5873489-15414078	10p15.1-p13
		chr10:15427950-26868173	10p13-p12.1
		chr10:27110043-27242311	10p12.1
		chr2:137963865-138787590	2q22.1
		chr2:112974505-113379867	2q14.1
		chr9:128309435-138124704	9q34.11-q34.3
		chr9:120763799-121365807	9q33.2
2G1-H4	chr2:142054244-181967534	chr20:16638826-25625984	20p12.1-p11.21
		chr20:142056-1467297	20p13
		chr20:31301793-59481159	20q11.21-q13.32
		chr20:59573167-64276082	20q13.32-q13.33
3A1-F2	chr3:0-98269003	chr8:74497265-86044133	8q21.12-q21.3
		chr8:63163338-66403590	8q12.3-q13.1
		chr3:148749748-149247516	3q24-q25.1
		chr3:168139317-183100677	3q26.2-q26.33
		chr4:121305166-140269076	4q27-q31.1
		chr21:9860388-10114639	21p11.2
		chr9:39883273-40101748	9p12-p11.2
		chr9:41363764-41621239	9p11.2
		chr9:63544183-63698999	9q13
		chr9:66274930-66379174	9q21.11
		chr13:33906643-40680076	13q13.2-q14.11
		chr3:149338029-168104318	3q25.1-q26.1
		chr4:160328589-162175360	4q32.1-q32.2
		chr4:150045231-160313119	4q31.3-q32.1
		chr1:147100246-158184951	1q21.1-q23.1
		chr1:93439600-96434413	1p22.1-p21.3
5pter-qter	chr5:0-151663745	chr7:77072929-93047200	7q11.23-q21.2
		chr7:102691476-105569647	7q22.1-q22.3
		chr7:150896031-157417511	7q36.1.-q36.3
		chr7:22562781-22733045	7p15.3
		chr7:99955218-102551307	7q22.1
		chr7:149931563-150262684	7q36.1
		chr2:26171597-28801596	2p23.3-p23.2
		chr18:683166-844067	18p11.32
		chr22:31626131-32115679	22q12.2-q12.3
		chr4:1115354-3874051	4p16.3
		chr4:4183016-88079035	4p16.3-q22.1
		chr1:89498675-93369782	1p22.2-p22.1
		chr4:559243-1058871	4p16.3
		chr12:131894446-132945956	12q24,33
		chr22:24805798-28760295	22q11.23-q12.1
		chr12:107931580-110048615	12q23.3-q24.11
		chr12:110050988-121059734	12q24.11-q24.31
		chr12:121109639-131852016	12q24.31-q24.33
		chr7:55951659-56116445	7p11.2
		chr7:67371788-72722850	7q11.21-q11.23
		chr7:73145680-76520510	7q11.23
		chr7:97968996-99631744	7q21.3-q22.1
		chr7:99955218-102551307	7q22.1
		chr7:115497-6732018	7p22.3-p22.1
		chr13:26210757-33680968	13q12.13-q13.2
6pter-qter	chr6:0-149546170	chr7:93101327-97872805	7q21.2-q21.3
		chr7:7093365-12492920	7p22.1-p21.3
		chr7:112498864-128461586	7q31.1-q32.1
		chr7:128671928-149886174	7q32.1-q36.1

		chr7:150335378-150863779	7q36.1
		chr7:23214537-33063634	7p15.3-p14.3
		chr7:55364682-55573516	7p11.2
		chr4:88257546-94351949	4q22.1-q22.3
		chr4:120041924-121273532	4q27
		chr1:67166227-67851415	1p31.3
		chr2:88002903-88874857	2p11.2
		chr2:70805311-86867996	2p13.3-p11.2
		chr2:68487905-70797831	2p13.3
		chr3:126006258-129319641	3q21.2-q21.3
		chr3:12897779-15118656	3p25.2-p25.1
		chr3:64032928-75273450	3p14.1-p12.3
		chr3:12360-12059043	3p26.3-p25.2
		chr3:12075139-12855873	3p25.2
		chr3:129376089-129895684	3q21.3-q22.1
		chr10:42782538-45674259	10q11.21-q11.22
		chr12:12900-2743910	12p13.33
		chr22:17084921-18176973	22qcen-q11.21
		chr12:7905547-9061868	12p13.31
		chr12:2793954-7543294	12p13.33-p13.31
		chr12:9748769-32384500	12p13.31-p11.21
8pter-qter	chr8:0-129106662	chr19:7112172-8069598	19p13.2
		chr13:102881565-114327455	13q33.1-q34
		chr8:397986-5501230	8p23.3-p23.2
		chr8:5510625-5890510	8p23.2
		chr8:5904979-6718069	8p23.2-p23.1
		chr13:51812906-52637583	13q14.3
		chr13:19410576-19553321	13q12.11
		chr8:36859024-42639769	8p11.23-p11.21
		chr8:29332949-36820056	8p12-p11.23
		chr8:8251254-9782907	8p23.1
		chr8:12721564-18101445	8p23.1-p22
		chr21:9594592-9809818	21p11.2
		chr4:162582872-189963502	4q32.2-q35.2
		chr8:18135309-20320465	8p22-p21.3
		chr19:16052230-19664128	19p13.12-p13.11
		chr22:33262346-35459147	22q12.3
		chr4:140330768-149971177	4q31.1-q31.23
		chr19:12627694-14572196	19p13.13-p13.12
		chr16:46659361-69942202	16q11.2-22.1
		chr16:70075624-74311815	16q22.1-q23.1
		chr1:146542324-146899900	1q21.1
		chr16:74408145-90043622	16q23.1-q24.3
		chr1:229229193-235161459	1q42.13-q42.3
		chr10:32752983-34863341	10p11.22-p11.21
10pter-qter	chr10:0-130495993	chr6:150073635-154676710	6q25.1-25.2
		chr6:122968765-149868599	6q22.31-25.1
		chr6:100098103-116726336	6q16.3-22.1
		chr6:116726514-122830290	6q22.1-22.31
		chr2:108449081-109621141	2q12.3-q13
		chr10:53435340-73103214	10q21.1-q22.1
		chr22:23054310-24635537	22q11.22-q11.23
		chr21:43939787-46665000	21q22.3
		chr19:14941357-15152099	19p13.12
		chr19:281181-4172053	19p13.3
		chr12:103965531-107783160	12q23.3
		chr22:32387312-33076428	22q12.3

		chr12:54962426-103957729	12q13.2-q23.3
11A1-B2	chr11:3207002-60782793	chr22:28772603-31626130	22q12.1-q12.2
		chr7:43924481-53156756	7p13-p12.1
		chr7:53191518-55250238	7p12.1-p11.2
		chr2:53660668-68467594	2p16.2-p13.3
		chr16:28819-173821	16p13.3
		chr5:173309722-174284250	5q35.2
		chr5:154952277-172505309	5q33.2-q35.1
		chr5:178104362-180869289	5q35.3
		chr5:131159221-134727937	5q23.3-q31.1
		chr5:151002149-154951429	5q33.1-q33.2
		chr1:227732052-228515511	1q42.13
		chr17:17013944-19306907	17p11.2
11D-E2	chr11:98845630-121935727	chr17:40779710-45561456	17q21.2-q21.31
		chr17:45629380-47073225	17q21.31-21.32
		chr17:47074949-47441070	17q21.32
		chr7:128466497-128616266	7q32.1
		chr17:62298466-64764269	17q23.2-q24.1
		chr17:64952801-68114549	17q24.1-q24.2
		chr17:68228066-83227287	17q24.2-q25.3
13A1	chr13:3323863-8582741	chr10:92758-4603604	10p15.3-p15.1
13C2-D2	chr13:79457838-119475736	chr5:88504238-96808680	5q14.3-q15
		chr5:50274162-85076091	5q11.2-q14.3
		chr1:121407541-121606937	1p11.2
		chr5:43118864-45589259	5p12
15D1-D3	chr15:64103833-72734561	chr8:130661023-136853592	8q24.22-q24.23
		chr8:136870681-140569867	8q24.23-q24.3
15D1-F1	chr15:63728908-96200954	chr8:130286098-136853592	8q24.21-q24.23
		chr8:136870681-144619253	8q24.23-q24.3
		chr22:35566904-50784010	22q12.3-q13.33
		chr12:33053427-34105230	12p11.21-cen
		chr12:38213339-47251325	12q12-q13.11
16C2-C4	chr16:69980809-94448349	chr3:84708526-90260450	3p12.1-cen
		chr2:131846708-132000018	2q21.2
		chr21:14143207-38498455	21q11.2-q22.2
19C1-D1	chr19:28764464-47748722	chr9:5235894-6659223	9p24.1
		chr10:50157843-52780322	10q11.23-q21.1
		chr10:87474356-106112500	10q23.2-q25.1
XA1-A2	chrX:5413066-24072117	chrX:48455475-51616130	Xp11.23-p11.22
		chrX:37491877-47660779	Xp21.1-p11.3
		chrX:115988254-118451148	Xq23-q24
XB-E1	chrX:81752585-111066899	chrX:24791056-37457604	Xp21.3-p21.1
		chrX:63633840-82146311	Xq11.2-q21.1
	Loss of Copy Numbers		
1F-G	not annotated	n.a.	n.a.
4C3-D2	chr4:72690706-121722416	chr9:81748815-83600221	9q21.32
		chr9:6847129-27300708	9p24.1-p21.2
		chr1:58654679-67096416	1p32.1-p31.3
		chr1:933238-24013566	1p36.33-p36.11
7D2-F5	chr7:75074861-145321697	chr15:98238393-98534827	15q26.3
		chr15:85286426-90272383	15q25.3-q26.1
		chr15:90315611-91022682	15q26.1
		chr15:82536714-85139183	15q25.2-q25.3
		chr15:79976352-82285402	15q25.1-q25.2
		chr11:49228782-49787688	11p11.12
		chr11:71915986-89617733	11q13.4-q14.3

		chr11:3609839-17345978	11p15.4-p15.1
		chr16:16587733-18231333	16p13.11-p12.3
		chr16:21561434-28327315	16p12.2-p12.1
		chr16:28379524-29010081	16p12.1-p11.2
		chr16:29645497-31509427	16p11.2
		chr10:119465080-133482234	10q26.11-q26.3
		chr11:192898-3233439	11p15.5-p15.4
		chr11:68960674-71501928	11q13.3-q13.4
9A1-B	chr9:3046525-56453749	chr11:90127365-107565913	11q14.3-q22.3
		chr19:8808332-11579065	19p13.2
		chr7:33094750-36454430	7p14.3-p14.2
		chr11:107581891-135035473	11q22.3-q25
		chr15:51057425-51650305	15q21.2
		chr15:51669611-59465398	15q21.2-q22.2
9E3-F4	chr9:97679785-121889089	chr3:146861495-148369705	3q24
		chr3:130212792-138634516	3q22.1-q22.3
		chr3:46394232-52312371	3p21.31-p21.2
		chr3:27712199-37219649	3p24.1-p22.2
		chr3:37227752-44140545	3p22.2-p21.32
11C-11D	chr11:85869344-95961140	chr17: 50794208-60886004	17q21.33-17q23.2
12pter-qter	chr12:0-120003175	chr2:10162883-26139074	2p25.1-p23.3
		chr2:9214594-10144790	2p25.1
		chr2:172258-9138189	2p25.3-p25.1
		chr7:105569791-108131740	7q22.3-q31.1
		chr7:12522126-19709187	7p21.3-p21.1
		chr7:108131761-112496091	7q31.1
		chr2:94607466-94720140	2qcen
		chr9:40364174-40478572	9p11.2
		chr9:42990554-43106968	9qcen
		chr9:64504248-66012148	9q13-q21.11
		chr14:24687986-51784456	14q12-q22.1
		chr14:58199894-105939623	14q23.1-q32.33
		chr7:157432951-159145209	7q36.3
		chr7:19713790-22489274	7p21.1-p15.3
14pter-qter	chr14:7756543-124743915	chr3:57956447-64024024	3p14.3-p14.1
		chr3:23104895-27667234	3p24.3-p24.1
		chr14:51805337-52106937	14q22.1
		chr6:39101990-39298710	6p21.2
		chr10:73110406-79495343	10q22.2-q22.3
		chr10:80053353-80210587	10q22.3
		chr3:52316044-57945503	3p21.1-p14.3
		chr3:15203607-16266323	3p25.1
		chr10:45868532-49967632	10q11.22-11.23
		chr10:80261810-87216559	10q23.1-q23.2
		chr14:52221917-58163176	14q22.1-q23.1
		chr14:19735448-22100195	14q11.2
		chr14:22132047-24680753	14q11.2-q12
		chr13:24761821-24937784	13q12.12-q12.13
		chr13:19633139-22705235	13q12.11-q12.12
		chr13:49247854-49587268	13q14.2
		chr13:25025580-26075099	13q12.13
		chr13:23279259-24322217	13q12.12
		chr13:49620101-51782351	13q14.2-q14.3
		chr8:9812600-11884194	8p23.1
		chr8:20349073-29293682	8p21.3-p12
		chr13:40895805-49224923	13q14.11-q14.2
		chr13:52651898-102437231	13q14.3-q33.1

15A1-B3	chr15:10872962-34071287	chr5:21002445-42888873<br>chr8:96434404-100628477	5p14.3-p12<br>8q22.1-q22.3
16B5-C2	chr16:45398818-69968181	chr3:108061366-125624615<br>chr3:75817777-84695898	3q13.12-q21.2<br>3p12.3-p12.1
18E2-E4	chr18:70643279-90560755	chr18:47241490-56577588<br>chr18:68672524-80209980	18q21.1-q21.31<br>18q21.1-q23
All Breakpoints Observed			
1A4	chr1:20494109	chr6:51899790	6p12.2
1A5	chr1:29328338	chr6:68127998	6q12
1B	chr1:35737073	chr2:130646039	2q21.1
1C1	chr1:52112433	chr2:194479680	2q32.3
1F	chr1:145557066	chr1:178469068	1q25.2
1H2	chr1:160649380	chr1:193561382	1q31.2
1H5	chr1:182502146	chr1:214738082	1q41
2B	chr2:32293689	chr9:121365807	9q33.2
2G1	chr2:142054244	chr20:16638826	20p12.1
3A3	chr3:23455115	chr3:171004698	3q26.2
3B	chr3:38265128	chr4:126317020	4q28.1
3F2	chr3:98269003	chr1:96434413	1p21.3
4C3	chr4:72690706	chr9:81748815	9q21.32
4D2	chr4:121722416	chr1:24013566	1p36.11
7D2	chr7:75074861	chr15:98238393	15q26.3
8B3	chr8:66146446	chr4:188712603	4q35.2
8C2	chr8:80082358	chr4:146587306	4q31.22
8C5	chr8:94642499	chr16:57025726	16q13
9B	chr9:56453749	chr15:59465398	15q22.2
9E3	chr9:97679785	chr3:146861495	3q24
9F1	chr9:105995278	chr3:138578062	3q22.3
9F4	chr9:121889089	chr3:44140545	3p21.33
10A1	chr10:7576744	chr6:123796551	6q22.31
10A3	chr10:23524569	chr6:139744376	6q24.1
10B5	chr10:74415065	chr10:72605253	10q22.1
10D2	chr10:118198336	chr12:91793538	12q21.33
11B2	chr11:60782793	chr17:19306907	17p11.2
11C	chr11:85869344	chr17:50794208	17q21.33
11D	chr11:98845630	chr17:40779710	17q21.2
11D	chr11:95961140	chr17:60886004	17q21.2
11E2	chr11:121935727	chr17:83227287	17q25.3
12F1	chr12:109851393	chr14:102074465	4q32.31
13C2	chr13:79457838	chr5:88504238	5q14.3
13D2	chr13:119475736	chr5:45589259	5p12
15A1	chr15:10872962	chr5:21002445	5p14.3
15B3	chr15:34747174	chr8:101304364	8q22.3
15D1	chr15:64103833	chr8:130661023	8q24.22
15D3	chr15:72734561	chr8:140569867	8q24.3
15F1	chr15:96200954	chr12:47251325	12q13.11
16B5	chr16:45398818	chr3:108061366	3q13.12
16C2	chr16:69980809	chr3:84708526	3p12.1
16C4	chr16:94448349	chr21:38498455	21q22.2
17B1	chr17:36006878	chr6:31996284	6p21.33
17B3	chr17:43727265	chr6:43499005	6p21.1
18E2	chr3:108061366	chr18:47241490	18q21.1
19C1	chr19:28764464	chr9:5235894	9p24.1
19D1	chr19:47748722	chr10:106112500	10q25.1
XA2	chrX:24072117	chrX:118451148	Xq24

XA4	chrX:45962571	chrX:125648865	Xq25
XA7	chrX:73826146	chrX:153512440	Xq28
XB	chrX:81752585	chrX:24791056	Xp21.3
XE1	chrX:111066899	chrX:82146311	Xq21.1
